# The Basolateral Amygdala Mediates the Role of Rapid Eye Movement Sleep in Integrating Fear Memory Responses

**DOI:** 10.3390/life12010017

**Published:** 2021-12-23

**Authors:** Mayumi Machida, Brook L. W. Sweeten, Austin M. Adkins, Laurie L. Wellman, Larry D. Sanford

**Affiliations:** Center for Integrative Neuroscience and Inflammatory Diseases, Pathology and Anatomy, Sleep Research Laboratory, Eastern Virginia Medical School, Norfolk, VA 23507, USA; machidmva@gmail.com (M.M.); bsweeten@tulane.edu (B.L.W.S.); adkinsam@evms.edu (A.M.A.); wellmall@evms.edu (L.L.W.)

**Keywords:** amygdala, fear memory, optogenetics, sleep, stress

## Abstract

The basolateral amygdala (BLA) mediates the effects of stress and fear on rapid eye movement sleep (REM) and on REM-related theta (θ) oscillatory activity in the electroencephalograph (EEG), which is implicated in fear memory consolidation. We used optogenetics to assess the potential role of BLA glutamate neurons (BLA^Glu^) in regulating behavioral, stress and sleep indices of fear memory, and their relationship to altered θ. An excitatory optogenetic construct targeting glutamatergic cells (AAV-CaMKIIα-hChR2-eYFP) was injected into the BLA of mice. Telemetry was used for real-time monitoring of EEG, activity, and body temperature to determine sleep states and stress-induced hyperthermia (SIH). For 3 h following shock training (ST: 20 footshocks, 0.5 mA, 0.5 s, 1 min interval), BLA was optogenetically stimulated only during REM (REM + L) or NREM (NREM + L). Mice were then re-exposed to the fear context at 24 h, 48 h, and 1 week after ST and assessed for behavior, SIH, sleep and θ activity. Control mice were infected with a construct without ChR2 (eYFP) and studied under the same conditions. REM + L significantly reduced freezing and facilitated immediate recovery of REM tested at 24 h and 48 h post-ST during contextual re-exposures, whereas NREM + L had no significant effect. REM + L significantly reduced post-ST REM-θ, but attenuated REM-θ reductions at 24 h compared to those found in NREM + L and control mice. Fear-conditioned SIH persisted regardless of treatment. The results demonstrate that BLA^Glu^ activity during post-ST REM mediates the integration of behavioral and sleep indices of fear memory by processes that are associated with θ oscillations within the amygdalo-hippocampal pathway. They also demonstrate that fear memories can remain stressful (as indicated by SIH) even when fear conditioned behavior (freezing) and changes in sleep are attenuated.

## 1. Introduction

The basolateral amygdala (BLA) plays a critical role in the consolidation of fear memory and in fear memory extinction [1,2,3,4,5] as indicated by findings that plasticity associated with learning and storage of conditioned fear occurs within BLA [6]. BLA has extensive reciprocal connections with the hippocampus [7,8], which is thought to provide contextual information during fear learning [9] and extinction [6]. BLA also is a major target for the medial prefrontal cortex (mPFC) [7,10], which is thought necessary for the expression of fear and for the consolidation of extinction memory [11]. Most of what is known about fear circuitry is based on regulation of overt fear behaviors in wakefulness, most prominently freezing [12,13,14,15]. However, we have found that BLA also plays an important role in fear-associated changes in sleep after fearful experiences and the recall of fearful memories. Rapid eye movement sleep (REM), in particular, can be fear conditioned, and several studies in mice [16] and rats [17,18] have demonstrated that REM can be altered by both shock training (ST) and fearful memories alone, and that fear-induced alterations in REM are regulated by BLA [19,20,21].

Recently, we demonstrated that brief optogenetic inhibition of BLA glutamatergic neurons (BLA^Glu^), specifically for 10 sec around the time of shock presentation, could attenuate ST-induced reductions in REM without altering subsequent fear memory-induced freezing [16]. We further found that, under baseline conditions, optogenetic inhibition of BLA^Glu^ during naturally occurring REM enhances REM-associated-theta (θ) activity in the EEG (REM-θ), increases attempts at transitioning into REM, and thus can increase REM amount under some circumstances [16]. By comparison, optogenetic activation of BLA^Glu^ during spontaneous REM reduces REM-θ without otherwise altering REM amounts or propensity for REM [22].

EEG θ activity is thought to reflect hippocampal θ oscillations [23], which functionally and temporally connect the hippocampus, BLA and mPFC [24,25,26,27,28]. These oscillations have been hypothesized to serve as a means to enable network-level cooperation for the collective actions of single neuron computations underlying cognitive functions [26,27], including memory consolidation [29], and are associated with the organization and initiation of motor activity [30]. REM-specific hippocampal θ oscillations have been suggested to be necessary for the consolidation of contextual fear memory [29]. The initiation and expression of fear states involve synchronized θ activity in the hippocampus, mPFC and BLA [24,31,32,33] and reduced correlations of θ activity in these regions has been associated with fear extinction [24]. However, we recently found that extinction at 24 h and 48 h after ST was associated with similar freezing and REM amounts, but with differences in other overt behaviors, in stress-induced hyperthermia (SIH), and in REM-θ [34]. Specifically, periods of reduced REM-θ were found immediately after and during the subsequent dark period when fear recall was conducted at 24 h after ST. By comparison, REM-θ did not significantly differ from baseline levels when extinction was conducted at 48 h after ST.

These results suggest that REM-θ may play a role in coordinating behavioral and physiological elements of fear expression and that BLA may influence links between overt behaviors and fear-induced alterations in REM potentially by impacting θ activity. To test this possibility, we optogenetically activated BLA^Glu^ of mice selectively during REM immediately after ST, and within the time period critical for fear memory consolidation [35,36]. We then evaluated the effects of post-ST REM-BLA^Glu^ activation on subsequent fear memory as measured by freezing, by fear conditioned changes in REM and REM-θ, and by fear conditioned stress responses as indicated by SIH.

## 2. Materials and Methods

### 2.1. Subjects

Male C57BL/6NCrl (B6) mice were obtained from Charles River (Wilmington, MA). The mice were 8–9 weeks old and weighed 20–25 g at arrival. The animals were individually housed and kept in a colony room with food and water available, ad libitum. The colony room was maintained on a 12:12 light-dark cycle and ambient temperature at 24°C ± 1.5 °C. Throughout the experimental procedures, measures were taken to minimize unnecessary pain and discomfort of the animals. All procedures were conducted in accordance with the National Institutes of Health Guide for the Care and Use of Experimental Animals and were approved by Eastern Virginia Medical School’s Institutional Animal Care and Use Committee, Protocol 17-015, approved 15 September 2017.

### 2.2. Virus Vector Construct

Purified adeno-associated virus preparations (AAV_5_) containing CaMKIIα-hChR2 (E123A)-eYFP-WPRE (ChR2), or CaMKIIα-eYFP-WPRE (eYFP) were obtained from the UNC Virus Vector Core Facility (University of North Carolina at Chapel Hill). The final viral concentration for injection was 1.5 × 10^12^ virus molecules/mL in 350 mM NaCl, 5% D-Sorbitol.

### 2.3. Surgical Procedures

Surgical procedures were conducted one week after arrival, during the light period with the mice under isoflurane anesthesia as inhalant (5% induction; 2% maintenance). The animals received prophylactic potassium penicillin (25 IU/g), gentamicin (0.005 mg/g) and dexamethasone (0.0005 mg/g) administered subcutaneously, and they were implanted intraperitoneally with telemetry transmitters (ETA-F10 or ETA-F20, Data Sciences International (DSI), St. Paul, MN, USA) to measure the electroencephalograph (EEG), activity and temperature. Electrode leads from the transmitter body were led subcutaneously to the head, and the free ends were placed into holes drilled in the skull (approximate coordinates from Bregma: 1.5/−3.5 mm AP, ±1.5 mm ML) to record cortical EEG. Microinjection cannulae were stereotaxically lowered bilaterally into BLA (−1.5 mm AP, ±2.9 mm ML, −4.7 mm DV) for administration of constructs containing either ChR2, or eYFP only for control experiments. We used 26-gauge injector cannulae connected to a syringe pump (BSP-99M, Braintree Scientific Inc., Braintree, MA, USA) at a flow rate of 0.1 µL/min. Each injection delivered 0.5 µL of virus vector, 1 µL total per animal. The cannulae remained in place for an additional 5 min to allow diffusion of viral particles away from the injection site. Custom-made optic probes (200 µm, conical tip, mated to metal or ceramic ferules) were then implanted directly above the injection site in BLA and secured to the skull using dental cement. Ibuprofen (30 mg/kg, oral) was continuously available in each animal’s drinking water for 24–48 h pre-operatively and for a minimum of 72 h post-operatively to alleviate potential operative pain. The animals were given 4 weeks for post-surgery recovery and to allow for viral expression. During the recovery period, the animals were kept undisturbed except for weekly cage changes.

### 2.4. Experimental Procedures

**Figure 1:**
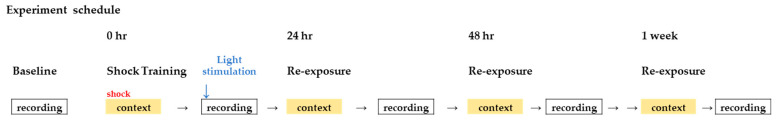
Schematic of the experimental procedure used in the study. Initially undisturbed electroencephalograph (EEG) recording was obtained and used as a baseline. All mice were then contextually fear conditioned through shock training (ST). Immediately after conditioning, mice received light stimulation during rapid eye movement sleep (REM), or non-rapid eye movement sleep (NREM), whereas control mice received no light. Twenty-four h, 48 h and 1 week after ST, mice were re-exposed to context alone. Recordings were conducted after each manipulation.

Methods for fear conditioning and contextual memory testing have been described in Machida et al. [37] and the experimental outline is provided in Figure 1. After recovery from surgery, undisturbed recordings were obtained for each animal and used as baseline. Animals were then habituated for optogenetic stimulation by being tethered with bilateral, flexible 45 cm-long optic fibers coupled to the implanted optic probes for 2 consecutive days prior to beginning the experiment. All experimental procedures started at the 6th hour after lights on and were conducted in a behavior test room adjacent to the colony room. Mice were contextually fear conditioned using shock training (ST) in which footshocks (0.5 mA, 0.5 sec duration, 20 trials, 1 min inter-trial interval) were administered via grid floors by Coulbourn Precision Regulated Animal Shockers (Model E13-14, Coulbourn Instruments, Whitehall, PA, USA). The conditioning procedure lasted for 30 min and consisted of a 5 min pre-ST period, 20 min ST period, and a 5 min post-ST period. Twenty-four h, 48 h, and 1 wk after ST, the animals were re-exposed to the shock chamber for 30 min without shock being presented. The behavior of the mice during the re-exposure was videotaped for subsequent scoring of freezing.

### 2.5. Optogenetic Stimulation

Optogenetic ChR2-mediated activation was produced through optic probes implanted in BLA using blue light (473 nm). ChR2 has been used to optically stimulate cells; upon illumination with blue light, ChR2, a cation channel, is opened allowing an inward current to depolarize the cell membrane within 50 µs of illumination [38].

Light output was measured by an optical power meter and adjusted to ~10 mW at the optic fiber tip at the frequency of 20 Hz (20-ms light pulses) to manipulate neurons at physiologically relevant frequencies in BLA [39]. Light was produced by a hybrid multi LED driver/fiber rotary joint (Part# D430-1106 (Blue/Green), Doric Lenses Inc., Quebec, Canada). Groups of ChR2-mice were manually presented light stimuli during REM (*n* = 7) and a separate group of ChR2-mice was administered light during NREM (*n* = 5). A separate group of eYFP-mice (*n* = 8) received ST and were recorded for controls.

### 2.6. Sleep Scoring

Immediately after conditioning, the mice were returned to their colony room. Each cage was placed on a telemetry receiver (RPC-1, DSI), and EEG, core body temperature, and a transistor-transistor logic (TTL) pulse from the transmitter were processed and collected by a DSI software. TTL pulses generated when the mice moved around in their cages were used as a measure of activity. Behavioral states of the animals were determined visually by a trained observer based on EEG and activity in 10 sec epochs using a scoring program (SleepSign for Animal, Kissei, Nagano, Japan) as previously described [37,40]. Briefly, each epoch was scored either as active wakefulness (AW, with movement recorded in epoch), quiet wakefulness (QW, no activity during epoch), NREM or REM. During NREM sleep, the EEG was characterized by high amplitude, slow waves, while REM was characterized by regularly spaced, lower amplitude waves and increased theta activity.

### 2.7. Electroencephalograph (EEG) Analysis

EEG signals were digitized at 256 Hz and spectrally analyzed by fast Fourier transformation (FFT) using the SleepSign program. The overall power in spectral bands between 0–4 Hz and between 5–8 Hz was summed and defined as delta wave amplitude (DWA) to represent delta activity, or theta wave amplitude (TWA) to represent theta activity, respectively.

### 2.8. Scoring of Freezing

Freezing, defined as a rigid posture with the complete absence of visible movement except for respiration, has frequently been used to evaluate degree of fear and retention of fear memory [41]. Freezing was scored by a trained observer for 5 min before and after ST, and immediately after exposure to the context. The percentage time spent freezing was calculated for each animal and expressed as mean ± SEM.

### 2.9. Evaluation of Body Temperature

SIH (also called psychogenic fever) is a stress-induced increase in body temperature that occurs in all mammals in preparation for fight-or-flight reactions. SIH occurs within 10 s of being exposed to a stressor and it has a time course that parallels that of HPA axis activation, and thus has been used as a measure of acute stress response [42]. It was evaluated for 4 h immediately after ST and after context re-exposures as a measure of the initial stress response and as an index of conditioned stress, respectively. SIH was defined as the difference in body temperature between stress-induced conditions (T_S_) and basal (T_B_) measurements (ΔT = T_S_ − T_B_) [42]. Under basal conditions, the mean core body temperature of the experimental ChR2 and control eYFP mice did not significantly differ.

### 2.10. Statistical Analysis

The data were analyzed using Sigma Plot 12.0 (Systat Software, Inc., San Jose, CA, USA) with between groups and repeated measures analysis of variance (ANOVA) procedures. In cases where the equal variance test failed, the data were analyzed using the Kruskal–Wallis ANOVA for ranks. When appropriate, post hoc comparisons were conducted using Tukey tests and differences were considered significant at *p* < 0.05. Two animals were presented with insufficient intensity footshock and were excluded from the study.

## 3. Results

### 3.1. Histological Verification of Stimulation Sites

**Figure 2:**
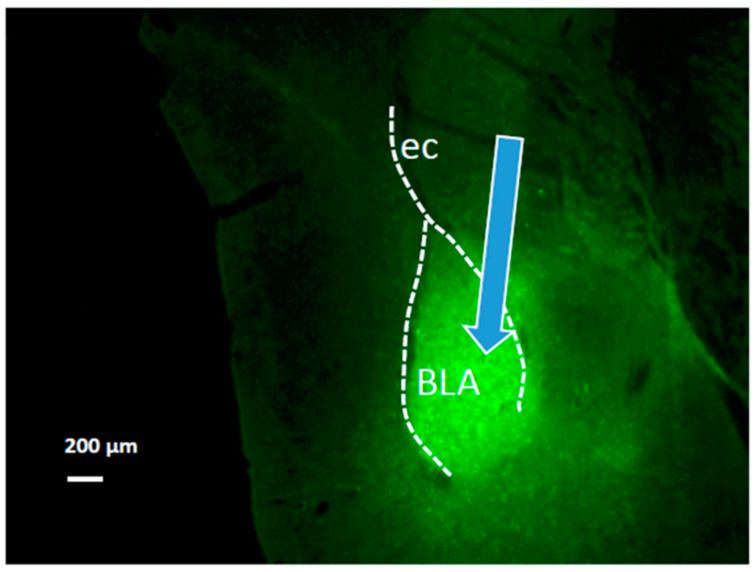
Viral constructs were stereotaxically injected into basolateral amygdala (BLA). Expression of ChR2-eYFP (green) is demonstrated in a representative coronal section of BLA (Bregma −2 mm 70). Blue arrow indicates optic probe location. Dotted line indicates external capsule (ec). Image (10×) was obtained using a fluorescent microscope (Nikon Eclipse E800, with yellow GFP BP HYQ filter; Nikon Instruments Inc., Melville, NY, USA). Scale bar, 200 µm.

After the experimental protocol was completed, BLA was evaluated for opsin expression and localization of the optic probe for light delivery (see example in Figure 2). Mice showing no eYFP expression in BLA due to faulty microinjections and mice showing optic probe placements outside of BLA were excluded from the study.

### 3.2. Rapid Eye Movement Sleep (REM) + L, but Not Non-Rapid Eye Movement Sleep (NREM) + L, Attenuated Freezing Tested at 24 h and 48 h after Fear Conditioning

**Figure 3:**
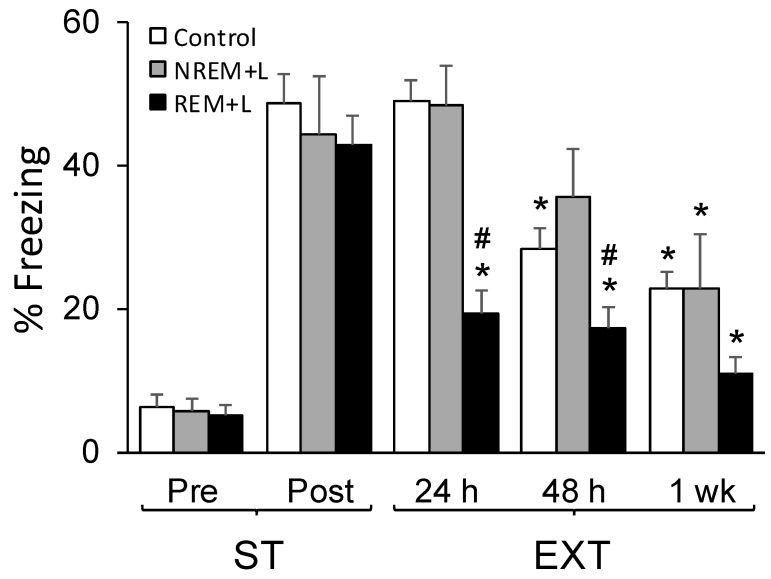
Effects of optogenetic activation on freezing of control, light stimulation during NREM (NREM + L) and during REM (REM + L). Freezing was scored for 5 min, before shock training (pre-ST), after ST (post-ST), and for 5 min immediately after re-exposed to the conditioned context at 24 h, 48 h and 1 week (wk) after conditioning. All data are represented as mean ± SEM. *: *p* < 0.05 compared to post-ST freezing. #: *p* < 0.05 compared to control and NREM + L.

Pre- and post-ST levels of freezing did not differ, thereby suggesting that behavioral fear levels prior to optogenetic stimulation were similar across groups (Figure 3). Within group ANOVAs comparing freezing across conditions were significant for the eYFP (F_(3, 21)_ = 10.804, *p* < 0.001), NREM + L (F_(1, 4)_ = 7.972, *p* = 0.048) and the REM + L (F_(3, 18)_ = 26.127, *p* ≤ 0.001) mice. In the eYFP control mice (Figure 3, white bars), freezing during the 1st re-exposure to the conditioned context (24 h) was almost identical to that exhibited post-ST, but it was reduced at the 48 h (*p* = 0.01) and 1 wk (*p* = 0.001) context re-exposures compared to post-ST levels. Mice in the NREM + L group (Figure 3, gray bars) also showed robust freezing at 24 h similar to post-ST levels, somewhat reduced levels at 48 h, and significantly reduced freezing compared to post-ST levels at the 1 wk context re-exposure. In contrast to the eYFP and NREM + L mice, optogenetic activation of BLA^Glu^ in the REM + L mice (Figure 3, black bars) showed reduced freezing compared to the post-ST level at the 24 h (*p* < 0.001), 48 h (*p* < 0.001), and 1 wk (*p* < 0.001) context re-exposures.

An ANOVA conducted to compare groups across time revealed a significant group effect (F_(2, 34)_ = 9.335, *p* = 0.002), time effect (F_(2, 34)_ = 25.349, *p* < 0.001) and a group X time interaction (F_(4, 34)_ = 3.037, *p* = 0.03,). Pairwise comparisons revealed that REM + L significantly attenuated freezing at 24 h (*p* < 0.001) and 48 h (*p* = 0.03) in comparison to the eYFP control mice and also at 24 h (*p* < 0.001) and 48 h (*p* = 0.026) in comparison to the NREM + L mice. At 1 wk after conditioning, the levels of freezing did not significantly differ among groups. The eYFP and NREM + L groups did not significantly differ at any time point. These results demonstrate that stimulation during REM alone had an impact on the subsequent expression of freezing.

### 3.3. Fear Conditioning and Stress-Induced Hyperthermia

Fear conditioning with footshock and subsequent re-exposure to the shock context alone resulted in SIH in all groups (Table 1). As seen in the eYFP control animals, compared to baseline, SIH was observed during H1 (F_(4, 28)_ = 40.256, *p* < 0.001) after ST (*p* < 0.001) as well as after context re-exposure at 24 h (*p* = 0.029), 48 h (*p* = 0.002), and 1 wk post conditioning (*p* = 0.015). SIH during H1 was also observed in the REM + L mice after ST (F_(1, 6)_ = 109.246, *p* < 0.001), and at 24 h (Χ^2^ = 7.000, *p* = 0.016), 48 h (F_(1, 6)_ = 52.613, *p* < 0.001) and 1 wk (F_(1, 6)_ = 62.000, *p* < 0.001) after context re-exposure (Table 1). Similarly, NREM + L mice (F_(4, 16)_ = 8.138, *p* < 0.001) showed SIH during H1 after ST (*p* < 0.001) and at 24 h (*p* = 0.005), 48 h (*p* = 0.013), and 1 wk (F_(1, 4)_ = 8.360, *p* = 0.045) after context re-exposure.

**Figure 4:**
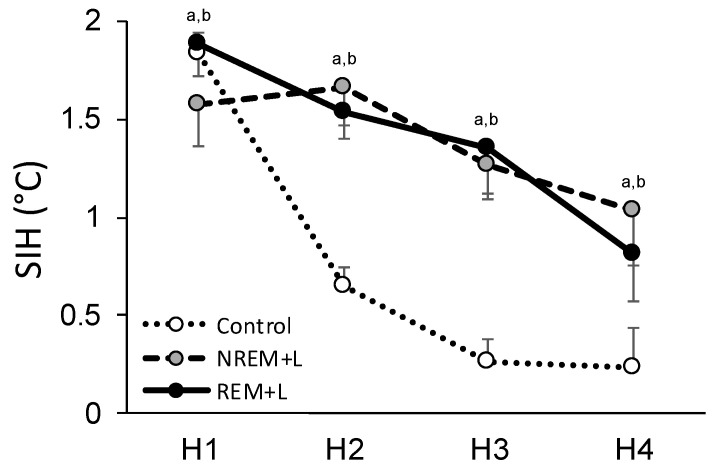
Effects of light stimulation on stress-induced hyperthermia (SIH) of control, NREM + L treatment and REM + L treatment mice across 4 h (H1–H4) following shock training (ST). All data are represented as mean ± SEM change from baseline. a: *p* < 0.05 compared to baseline for NREM + L. b: *p* < 0.05 compared to baseline for REM + L.

In agreement with previous work [16], we also found that procedures necessary for optogenetics (e.g., fiber connection to optic probe/tethering) can increase and extend the duration of SIH. This was indicted by the fact that we observed significantly increased temperature during H2–H4 in both the REM + L (H2: F_(1, 6)_ = 107.424, *p* < 0.001; H3: F_(1, 6)_ = 22.902, *p* = 0.003; H4: F_(1, 6)_ = 9.530, *p* = 0.021) and NREM + L (H2: NS; H3: F_(1, 4)_ = 72.446, *p* = 0.001; H4: F_(1, 4)_ = 13.565, *p* = 0.021) mice compared to baseline (Figure 4). There were no group differences in SIH after re-exposure to the context alone.

### 3.4. Stress and Fear-Conditioned Alterations in Sleep

**Figure 5:**
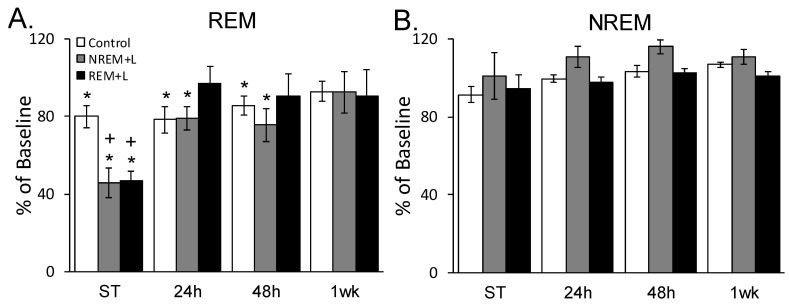
Effects of light stimulation on REM (**A**) and NREM (**B**) plotted as percentage baseline for 4 h immediately after shock training (ST) or successive context re-exposures (24 h, 48 h and 1 week (wk) post-training. Mean ± SEM. *: *p* < 0.05 in compared to baseline. +: *p* < 0.05 compared to control.

The ANOVA for REM revealed a significant group X time interaction (F_(6, 68)_ = 4.750, *p* < 0.001). A post hoc test found that 4 h totals of REM were significantly reduced compared to baseline in all groups after ST, and in the REM + L and NREM + L groups compared to the eYFP control group (Figure 5A). The greater ST-induced reductions in REM might be related to stress indicated by SIH, and not directly linked to activation of BLA^Glu^, given that REM sleep propensity is inversely proportional with body temperature [43]. Indeed, the correlation between hourly REM and SIH was high during REM + L (R^2^ = 0.93, *p* < 0.01) and relatively high during NREM + L (R^2^ = 0.67, NS).

REM was significantly reduced compared to baseline in the eYFP control and NREM + L groups after the 24 h and 48 h re-exposures, but returned to baseline levels after the 24 h context re-exposure in the REM + L group. REM returned to baseline levels in the eYFP control and NREM + L groups after the 1 wk context re-exposure. The 4 htotal NREM were not significantly altered, and there was no difference among groups (Figure 5B).

### 3.5. REM + L Attenuated Post-Re-Exposure REM-θ Reduction

**Figure 6:**
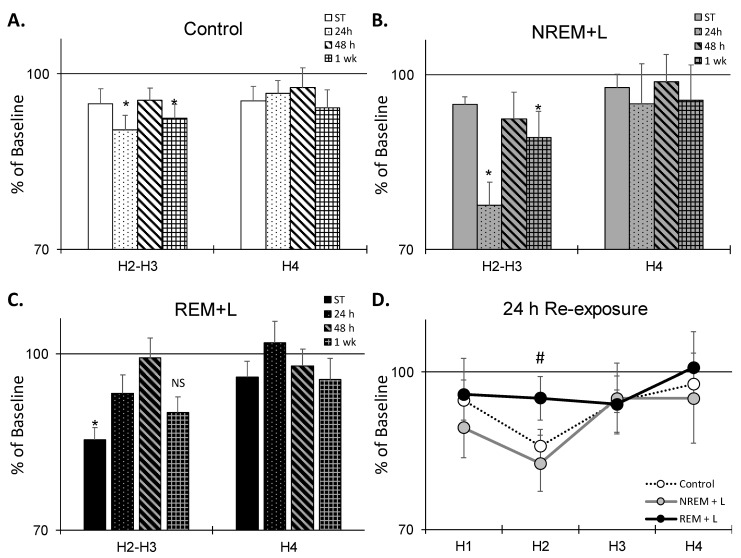
Effects of light stimulation on REM-θ wave activity (TWA) across days for no light control (**A**), NREM + L (**B**), and REM + L (**C**). Panel (**D**) shows comparison of effects of light stimulation across treatment conditions after the 24 h context re-exposure. All data are expressed as % of baseline. Mean ± SEM. *: *p* < 0.05 compared to baseline. #: *p* < 0.05 compared to Control and NREM + L. NS: nonsignificant.

Post-ST REM-θ was not significantly altered compared to baseline in either eYFP control (Figure 6A) or NREM + L (Figure 6B) mice whereas REM + L (Figure 6C) significantly attenuated post-ST REM-θ (H2–H3) in comparison to baseline (χ^2^ = 7, *p* = 0.016). In contrast, after the 1st context re-exposure (24 h), REM-θ was significantly attenuated in comparison to baseline in the eYFP control (F_(1, 7)_ = 22.193, *p* = 0.002), and in the NREM + L animals (F_(1, 4)_ = 8.320, *p* = 0.045), but *not* in the REM + L animals. This difference was due to a difference in REM-θ in the second h of recording after context re-exposure (Figure 6D). REM-θ was also significantly reduced compared to baseline after the 1 wk context re-exposure in the eYFP control (Figure 6A) NREM + L (Figure 6B) mice; however, the difference did not reach significance in the REM + L mice (Figure 6C).

## 4. Discussion

Our results found that stimulating BLA immediately after ST during REM significantly attenuated freezing tested 24 h after ST, compared to stimulation during NREM and non-stimulated eYFP control mice when fear was evoked by context re-exposures at 24 h after ST. REM + L also produced changes in fear conditioned REM responses. Significant reductions in REM were observed in the 4 h REM totals in the NREM + L and eYFP control mice. However, the REM + L mice showed a rapid recovery of REM indicated by almost baseline level of REM in the first 4 h after the 24 h and 48 h re-exposures. By comparison, SIH was similar across groups on each successive exposure to the fearful context. Thus, based on the conditioned stress response, the mice remembered the aversive context even though freezing and conditioned REM responses were attenuated.

Several lines of work now indicate that freezing, fear-induced changes in REM and SIH are dissociable measures of fear memory; they do not necessarily follow the same temporal course and are not mutually predictive. However, each can be fear-conditioned [17,34,44] and both freezing and REM responses can extinguish with subsequent non-reinforced presentation of the fearful stimulus [45]. They also can be dissociated experimentally and considerable evidence from studies manipulating BLA before, during and after ST, and prior to fear recall, demonstrate that it mediates the relationship between fear-conditioned behavioral and sleep responses [16,17,19,20,21]. The current work suggests that BLA-regulated influences on neural activity during post-ST REM may be critical for integrating responses as fear memory is consolidated. The attenuation of freezing and conditioned REM responses were marked even though post-ST REM amounts were reduced and stimulation of BLA was only conducted during H1–H3 after ST. By comparison, memory consolidation is thought to take place over up to 6 h after training [46].

We stimulated BLA at a frequency of 20 Hz which is within the frequency range (10–25 Hz) often used in studies of the amygdala and other regions [47,48,49,50], and was selected based on findings that 20 Hz stimulation of the lateral amygdala produces reliable and robust firing in pyramidal cells [51]. We also previously found that stimulation of BLA at 20 Hz attenuated REM-θ whereas optogenetic inhibition of BLA increased REM-θ [34]. It is possible that stimulation at other frequencies could have other effects on fear memory. For example, optogenetic stimulation of BLA at 40 Hz improves emotional memory [50], consistent with indications that neural activity in BLA within this range is important for emotional memory [52,53]. It has also been reported that optogenetic inhibition of medial septum GABA–releasing neurons during REM attenuated REM-θ without disturbing sleep, and subsequently impaired freezing [29], indicating that REM-θ may be modulated by other brain regions in addition to BLA.

While stimulation of BLA was conducted in the period after training, it also is possible that REM + L potentiated fear extinction when the mice were subsequently re-exposed to the fearful context alone. Fear memory and extinction are functionally different forms of associative emotional memory that have common neural circuitry and neuromodulation [54]. Although it is beyond the scope of the current study to distinguish the two forms of memory, we previously found that conditioned fear evoked at 24 h after ST was associated with behavior (rearing) that is thought to be reflective of reduced anxiety [55,56,57] and was followed by a temporally distinct attenuation of REM-θ amplitude. By comparison, older fear memory, evoked at 48 h post-ST, was not associated with increased rearing or with an attenuation of REM-θ [34]. Thus, BLA attenuation of REM-θ amplitude may have modulated the “fresh” fear memory in a manner similar to that observed during early extinction. Interestingly, after the 24 h context re-exposure, mice in the eYFP control and NREM + L groups showed attenuated REM-θ amplitude (primarily in the second h after context re-exposure) whereas mice in the REM + L group did not. This would be consistent with the hypothesis that the modulation of fear memory associated with attenuated REM-θ had already occurred.

We previously found that REM-specific optogenetic activation of BLA^Glu^ reduced REM-θ activity whereas optogenetic inhibition of BLA^Glu^ increased REM-θ [16,22]. Generally, large-scale θ oscillations detected in the EEG are thought to be primarily produced in the hippocampus [23], and changes in power amplitude are thought to result from macroscopic changes in synchronization within local neural ensembles [58]. Recently, θ oscillations have been suggested to enable network-level communication necessary for cognitive functions [26,27], including memory consolidation [29] and fear extinction [24], and in vivo hippocampal θ oscillations during REM have been hypothesized to be necessary for the consolidation of contextual fear memory [29]. Studies using local field potential (LFP) and unit recordings have found phase-locked discharge of neurons responding to hippocampal θ waves in the amygdala and the mPFC [29,59,60,61]. These structures exhibit “synchronized θ activity” reflecting selective involvement of the hippocampus [11], for example, θ coherence increases during contextual fear conditioning [33], but declines during extinction learning [24]. Synchronized θ activity has been hypothesized to provide a means for connecting neural ensembles temporally and functionally [24,25,26]. The BLA projects extensively to the hippocampus [7,8,62] and changes in REM-θ activity that were induced with optogenetic activation of BLA likely reflect its influence on local neural activity within the hippocampus [63].

It should be noted that the restraint and tethering required to implement optogenetic experiments can induce a stress response as indicated by SIH [16,22] which can, in turn, influence REM [43]. Indeed, we observed continued SIH and enhanced reductions in REM while mice were tethered during the post-ST REM + L and NREM + L treatment conditions. This prolonged period of stress and enhanced reduction of REM did not prevent the effects of REM + L on freezing nor subsequent normalization of REM evaluated at both 24 h and 48 h, This finding is at odds with hypotheses, based on selective REM deprivation experiments, that post-ST REM is of particular importance for the consolidation of both fear and extinction memory [35,36]. The finding that REM amount does not proportionally correlate with learning and memory is consistent with previous results from our lab (unpublished observations) [37] and others [64]. Indeed, REM can be either decreased or increased following virtually identical fear-induced freezing and stress responses [19,20,21,37,44], indicating that fear memory can differentially regulate outputs across functional systems. By comparison, REM-dependent physiological events including θ coherence [61], REM-θ amplitude [22,29] and phasic pontine-waves (P-waves) [65] may be more accurate predictors of successful consolidation of fear memory than REM amount. Interestingly, both REM-θ and P-waves [66,67,68] are regulated by the amygdala, which along with the mPFC and hippocampus, exhibits coordinated θ activity associated with contextual fear conditioning [33]. Thus, multiple aspects of neural activity in the circuitry underlying the processing of fear memory may have been impacted by activating BLA during REM. This may include effects on communication between the hippocampus, BLA and mPFC, as well as potentially other areas which play roles in fear memory.

Unfortunately, due to equipment limitations, we were not able to record temperature when the mice were in the shock context. Thus, our data reflect continuing increases in temperature after the mice had been returned to their home cages. However, these increases demonstrate that SIH had been induced by context re-exposure alone, indicating that it also was a fear-conditioned response. Its induction was not apparently impacted by optogenetic stimulation of BLA during REM, a finding consistent with other studies in which manipulations of BLA did not impact SIH [19,21]. Our results are also congruent with work in rats [19,20,21] and mice [34] that found that freezing can extinguish at high levels of SIH. Thus, neither attenuated freezing nor normalized REM may always indicate that a fearful memory has been extinguished and that it is no longer stressful.

In conclusion, the current study confirmed our prior finding that optogenetic activation of BLA^Glu^ specifically during post-shock REM reduced REM-θ amplitude [22] and provided additional support for the contention that REM-θ plays a functional role in the processing of fear memory [29]. The reduction in REM-θ amplitude was associated with subsequent attenuated freezing and altered fear-conditioned REM responses, whereas fear-conditioned SIH was not significantly impacted compared to other groups receiving identical ST and stimulation during NREM. These data demonstrate a significant role for BLA and REM-dependent processing in mediating some, but not all, aspects of fear memory. In general, the varying results for indices of behavioral fear, sleep and the stress response demonstrate the need to include multiple outcome measures in assessing fear memory and its association with sleep and stress, and in examining its neural regulation. This approach may lead to insight into the relationship between sleep and fear memory, and the complex repertoire of behavioral and physiological responses BLA regulates that is not possible with a single index alone.

## Figures and Tables

**Table 1:** Hourly change in temperature across recording days and experimental conditions.

Day	Hour	Control	NREM + L	REM + L
ST	H1	1.83 ± 0.11 *	1.57 ± 0.21 *	1.89 ± 0.17 *
	H2	0.64 ± 0.10	1.66 ± 0.19 *	1.54 ± 0.14 *
	H3	0.26 ± 0.12	1.27 ± 0.15 *	1.35 ± 0.26 *
	H4	0.24 ± 0.20	1.04 ± 0.28	0.82 ± 0.24 *
24 H	H1	1.54 ± 0.16 *	1.26 ± 0.19 *	1.51 ± 0.13 *
	H2	0.54 ± 0.09	0.74 ± 0.12	0.69 ± 0.17
	H3	0.35 ± 0.09	0.40 ± 0.17	0.30 ± 0.21
	H4	0.24 ± 0.11	0.06 ± 0.24	0.17 ± 0.10
48 H	H1	1.39 ± 0.16 *	1.11 ± 0.21 *	1.27 ± 0.16 *
	H2	0.35 ± 0.14	0.66 ± 0.10	0.71 ± 0.18
	H3	0.00 ± 0.08	0.26 ± 0.09	0.41 ± 0.22
	H4	0.06 ± 0.09	0.22 ± 0.16	0.28 ± 0.22
1 WK	H1	1.28 ± 0.19 *	0.86 ± 0.30 *	1.17 ± 0.14 *
	H2	0.29 ± 0.13	0.38 ± 0.11	0.45 ± 0.19
	H3	−0.01 ± 0.12	0.17 ± 0.17	0.00 ± 0.19
	H4	0.05 ± 0.09	0.01 ± 0.20	0.02 ± 0.25

ST: shock training day; 24 H: first context re-exposure; 48 H second context re-exposure; 1 week (WK): third context re-exposure. Control: no optogenetic stimulation; NREM + L: optogenetic stimulation during NREM; REM + L: optogenetic stimulation during REM. Data are presented as changes in temperature relative to time-matched baseline recordings. *, *p* < 0.05 compared to time-matched baseline.

## Data Availability

Experimental data available upon request.
